# Adopting a Mediterranean-style eating pattern with low, but not moderate, unprocessed, lean red meat intake reduces fasting serum trimethylamine N-oxide (TMAO) in adults who are overweight or obese

**DOI:** 10.1017/S0007114521004694

**Published:** 2022-11-14

**Authors:** Sridevi Krishnan, Lauren E. O’Connor, Yu Wang, Erik R. Gertz, Wayne W. Campbell, Brian J. Bennett

**Affiliations:** 1 Department of Nutrition, University of California-Davis, Davis, CA, USA; 2 Department of Nutrition Science, Purdue University, West Lafayette, IN, USA; 3 Division of Cancer Prevention, National Cancer Institute, National Institutes of Health, Rockville, MD, USA; 4 USDA-Western Human Nutrition Research Center, Davis, CA, USA

**Keywords:** trimethylamine N-oxide, Mediterranean diet, homoeostatic model assessment of insulin resistance, Framingham risk score, Vascular age

## Abstract

A Mediterranean-style eating pattern (MED-EP) may include moderate red meat intake. However, it is unknown if the pro-atherogenic metabolite trimethylamine N-oxide (TMAO) is affected by the amount of red meat consumed with a MED-EP. The results presented are from a secondary, retrospective objective of an investigator-blinded, randomised, crossover, controlled feeding trial (two 5-week interventions separated by a 4-week washout) to determine if a MED-EP with 200 g unprocessed lean red meat/week (MED-CONTROL) reduces circulating TMAO concentrations compared to a MED-EP with 500 g unprocessed lean red meat/week (MED-RED). Participants were seventy-seven women and twelve men (*n* 39 total) who were either overweight or obese (BMI: mean (30·5) (sem 0·3) kg/m^2^). Serum samples were obtained following an overnight fast both before (pre) and after (post) each intervention. Fasting serum TMAO, choline, carnitine and betaine concentrations were measured using a targeted liquid chromatography-MS. Data were analysed to assess if (a) TMAO and related metabolites differed by intervention and (b) if changes in TMAO were associated with changes in Framingham 10-year risk score. Serum TMAO was lower post-intervention following MED-CONTROL compared with MED-RED intervention (post-MED-CONTROL 3·1 (sem 0·2) µm *v*. post-MED-RED 5·0 (sem 0·5) µm, *P* < 0·001), and decreased following MED-CONTROL (pre- *v*. post-MED-CONTROL, *P* = 0·025). Exploratory analysis using mixed model ANCOVA identified a positive association between changes in TMAO and changes in homoeostatic model assessment of insulin resistance (*P* = 0·036). These results suggest that lower amounts of red meat intake lead to lower TMAO concentrations in the context of a MED-EP.

A Mediterranean-style eating pattern (MED-EP) is one of the healthy eating patterns recommended by the 2020–2025 Dietary Guidelines for Americans^(1)^. A consensus of what foods compose a MED-EP is lacking due to variations in food sources and preferences across Mediterranean regions^(2)^. However, a MED-EP is most often considered to contain a variety of fruits, vegetables, whole grains, nuts and legumes; olive oil as a primary source of fat; an emphasis on fish; moderate amounts of dairy product, red meat, poultry and egg; and red wine^(3)^. Results of PREDIMED, one of the largest studies to assess the health effects of consuming a MED-EP, showed a reduction in stroke, type 2 diabetes (T2D), peripheral arterial disease, atrial fibrillation and breast cancer incidence at a 4·8-year follow-up^(4)^. Several observational studies have assessed relations between MED-EP and CVD risk^(5–7)^. Inconsistences exist among the effect estimates from epidemiological studies and meta-analyses, albeit predominantly supporting cardio-protective effects of MED-EP.

In addition to associations between specific eating patterns such as the MED-EP, there is a plethora of research investigating associations between red meat intake and the risk of CVD. Most recent literature suggests that there is no association between unprocessed red meat intake and CVD risk, but a relatively strong association between processed red meat intake (up to 40 % increased risk) and certain CVD events or related death. T2D, a metabolic disease, is often associated with both unprocessed and processed red meat. Therefore, dietary recommendations often encourage reducing total and processed red meat intakes to reduce risk for cardiometabolic diseases, such as CVD and T2D^(8,9)^. Trimethylamine N-oxide (TMAO), a gut-microbiota-dependent metabolite^(10)^, has emerged as a potential mechanism to explain associations between red meat consumption and cardiometabolic disease. TMAO is largely derived from gut bacterial fermentation of dietary choline, carnitine and betaine to produce trimethylamine, which is further oxidised to TMAO by hepatic flavin monooxygenases^(11)^. Specific to meat intake, a randomised controlled trial has shown that red meat, but not white, increases circulating TMAO concentrations^(12)^. However, there have been limited randomised controlled trials that assess cause-and-effect relationships between meat intake and TMAO concentrations.

Currently, no study to our knowledge has investigated the effect of different amounts of red meat within the context of a MED-EP on plasma TMAO concentrations or its metabolite precursors choline, carnitine and betaine. Hence, the objectives of the current project were to (a) investigate if there were differential effects of two amounts of red meat fed within a background MED-EP on circulating TMAO and its metabolic precursors and (b) identify relationships between TMAO and related metabolites with clinical markers of cardiometabolic disease. To address these questions, we used archival fasting serum aliquots and data from a randomised, crossover feeding trial originally designed to assess the effects of consuming a MED-EP with 500 (MED-RED) *v*. 200 g/week (MED-CONTROL) of lean, unprocessed red meat on cardiometabolic disease risk factors^(13)^, 10-year lipid-based disease risk prediction^(13)^ and personal well-being^(14)^. We hypothesised that lower red meat consumption within a MED-EP would reduce TMAO and that TMAO would be associated with clinical outcomes such as Framingham 10-year risk score, and vascular age.

## Methods

### Experimental design and ethics

The experimental design was a randomised, crossover, investigator-blinded, controlled feeding study. Participants consumed a diet based on the MED-EP for two 5-week controlled feeding interventions, separated by at least 4 weeks of a self-selected and unrestricted pattern (washout). Aliquots of fasting serum were collected before (pre) and at the end (post) of the two intervention periods, and stored frozen at −80°C until analysed for compounds of interest. The study protocol and all study documents were approved by Purdue University’s Biomedical Institutional Review Board (institutional review board protocol 1501015662). All participants provided written informed consent and were provided monetary compensation. The testing phase of the study was conducted from July 2015 through December 2016, and the protocol is registered at clinicaltrials.gov (NCT02573129).

### Study participants

Forty-one adults (thirteen males and twenty-eight females) who were overweight or obese based on their BMI (25–37 kg/m^2^), between 30 and 69 years of age and were screened to ensure that they were not already consuming a MED-EP (using the 14-item Mediterranean Diet Assessment Tool^(15)^) completed both controlled feeding interventions. Inclusion/exclusion criteria are published^(13)^. In the current secondary analysis report, data from thirty-nine (twelve males and twenty-seven females) of the original forty-one participants were assessed.

### Diet intervention

The details of this intervention are published^(13)^. Briefly, menus were developed as described using ProNutra software (Viocare, Inc.) and followed the PREDIMED protocol to achieve the desired Mediterranean pattern. A detailed description of the nutrient and food composition of both interventions is described previously^(13)^. Fish and legume intake were similar in both Mediterranean patterns to achieve the desired eating pattern per the PREDIMED protocol. MED-RED and MED-CONTROL differed predominantly in the amounts of red meat and poultry provided but also in dairy product and grains to ensure that macronutrients were matched across the two interventions. Participants were given the option to consume 150 ml of self-selected dry red wine daily. Intervention-specific menus were designed to meet each participant’s estimated energy required to maintain body weight. For one of the two 5-week interventions, participants consumed a MED-EP containing a commonly recommended amount of lean red meat (∼200 g/week, MED-CONTROL), and the other 5-week intervention contained the average amount of red meat consumed in the USA (∼500 g/week, MED-RED).

All foods were prepared and provided to subjects during the two Mediterranean pattern interventions by the NIH-supported Indiana Clinical Research Center Bionutrition Facility at Purdue University. Participants were provided with all the foods and beverages (except wine) they were instructed to completely consume during the 5-week MED-CONTROL and MED-RED interventions. Subjects were weighed and met with study staff weekly to monitor body mass and promote compliance, respectively. Subjects completed daily (and returned weekly) menu check-off lists to track self-reported deviations from the provided Mediterranean pattern. Dietary intake and compliance were measured from the menu check-off lists of 3 d during the last week of each intervention.

### Liquid chromatography-MS analysis of trimethylamine N-oxide and related metabolites in serum

The metabolites TMAO, carnitine, betaine and choline were analysed from fasting serum samples via liquid chromatography-MS. The following methodology was adapted from Wang *et al*.^(16)^. In short, standards ranging from 0 to 100 µM of non-deuterated analytes in 1:1 of water:acetonitrile were employed to create a standard curve. To control for experimental drift, a 10 µm surrogate standard (SSTD) consisting of deuterated analytes in 1:1 water:acetonitrile was created. Twenty microlitre of each serum sample was combined with 80 µl SSTD; the mixture was vortexed for 30 s and centrifuged at 18 000 ***g*** at 10°C for 10 min. The supernatant was extracted and aliquoted into HPLC vials. Supernatants were acquired via Acquity UPLC (Waters) and measured via API 4000 Q-Trap (Applied Biosystems). The Acquity system was fitted with a silica column (4·6 by 250 mm, 5 µm Luna silica; Cat. No. 00G-4274-E0, Phenomenex) and guard column. A discontinuous gradient was generated to resolve the analytes by mixing solvent A (0·1 % propionic acid in water) with solvent B (0·1 % acetic acid in methanol) at different ratios starting from 2 % B linearly to 15 % B over 10 min, then linearly to 100 % B to 12·5 min, then after 3 min, back to 2 % B. Sample and extraction controls were employed. Known concentrations of human serum samples were run in triplicate at different time points in the experiment acting as sample controls; also, extraction controls were generated by duplicating the extraction protocol for multiple samples within an experiment.

### Cardiometabolic disease risk factors

Cardiometabolic disease risk factors were measured before and at the end of each intervention. Measurements included fasting serum lipids and lipoproteins (total cholesterol (total-C), LDL-cholesterol, HDL-cholesterol, total-C: HDL-cholesterol ratio, total ApoB, TAG, glucose, insulin and C-reactive protein; homoeostatic model assessment of insulin resistance (HOMA-IR); homoeostatic model assessment of pancreatic beta cell function (HOMA-B) and fasting and ambulatory blood pressures). Testing conditions and measurement methods were previously reported^(13)^. HOMA-IR was calculated as (fasting glucose mg/dl × fasting insulin µIU/ml)/405^(17)^ and HOMA-B was calculated using the formula: [(360 × insulin (µIU/ml))/(glucose(mg/dl)–63)] × 100. Cystatin C, a marker of kidney function, was measured in serum samples using a K-assay (Kamiya Biomedical Company), before and after both intervention periods. Poor kidney health could reduce the clearance of TMAO^(18)^, resulting in elevated concentrations in serum. Therefore, this was used to make sure none of the participants had kidney health issues that would confound TMAO outcomes.

### Framingham risk score and vascular age

Both Framingham risk score (FRS) and vascular age were calculated using age, biological sex, HDL-cholesterol, resting systolic blood pressure, smoking status, presence or absence of T2D and use of blood pressure medication using the 2017 version of the online calculator (https://framinghamheartstudy.org/fhs-risk-functions/cardiovascular-disease-10-year-risk/).

### Statistical analyses

#### Pre-preparation

The primary study power analyses, data management and statistical approaches are published^(13)^. For the current report, data obtained were screened for missing values. Missing data were imputed using multivariate normal, multiple imputation using least-squares prediction based on non-missing data in the array^(19)^. A total of 2·5 % of the data (195 values out of a total of 8520 in the array including all primary and secondary outcomes) were missing. Missing data were considered missing not at random (missingness is related to unknown observations/aspects)^(20)^. Data were then assessed for normal distribution using QQ plots, and Shapiro Wilk tests, and when deemed not normal, were converted to log or Johnson transformed versions to achieve normality^(21)^. Johnson distributions are a family of probability distributions that include the log-normal (SL), bounded (Sb) and unbounded (Su) transformations of the normal distribution. This family of distribution offers flexible exponential, logistic and hyperbolic sine transformations within the same function, making it a compelling alternative to use when log transformations do not succeed in normalising the data. Cauchy’s and Huber’s tests were used to look for outliers, and none was detected in transformed data that were used in final analyses. All analyses were done in R statistical software (version 3.6.0) and JMP Pro 15.1 (SAS Institute).

#### Effect of intervention on trimethylamine N-oxide and related metabolites

To address the question of whether there was a differential effect of the intervention periods on TMAO and related metabolite concentrations, the following analyses were done. Imputed data were used in linear mixed model analyses to assess if there was a carryover effect of treatment on TMAO, betaine, choline and carnitine by including a sequence × treatment interaction term. To assess the primary fixed effect of the treatment on each of these metabolites, linear mixed models were developed. Specifically, treatment period (MED-CONTROL *v*. MED-RED), time (pre *v*. post), sex (male *v*. female) and their interaction (treatment × time, sex × treatment, sex × time, sex × treatment × time) were modelled as fixed effects, and participant as the random effect and Cystatin-C was used as a covariate. Tukey’s multiple comparison-adjusted *P*-value estimations were calculated when there was a significant treatment × time, treatment × sex or treatment × sex × time interaction for any outcome assessed using linear mixed models; *P*-values < 0·05 were considered significant.

#### Exploratory analysis between trimethylamine N-oxide and clinical parameters

To address the second part of our objective, we assessed whether TMAO was associated with HOMA-IR and HOMA-B or CVD risk (FRS or vascular age). Imputed data were used for all exploratory analyses. We used a mixed model ANCOVA to verify the robustness of identified associations. We assessed normalised (mean 0 and (sd 1)) TMAO, choline, betaine and carnitine as covariates in individual models with fixed (treatment, time, treatment × time interactions) and random effects (subject, subject × time, subject × treatment and subject × covariate interactions) predicting FRS, vascular age, HOMA-B or HOMA-IR.

## Results

The participant characteristics are published^(13)^. Briefly, participants were generally healthy (based on cardiometabolic clinical risk measures), overweight or obese (BMI: mean 30·5 (sem 0·6) kg/m^2^) and between 30 and 69 years of age (age: mean 46 (sem 2) years).

### Intervention effect on trimethylamine N-oxide and its metabolic precursors

**Fig. 1.**
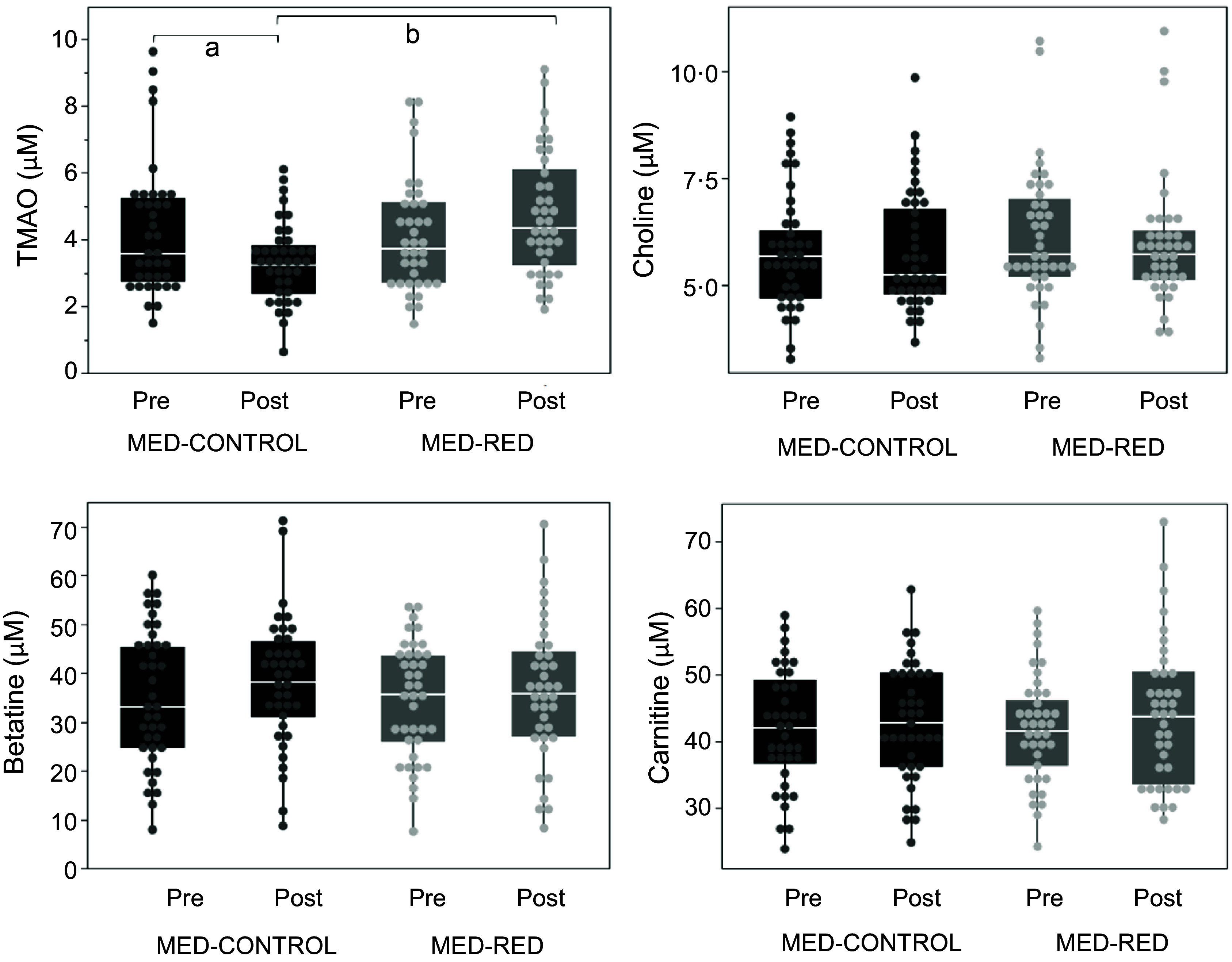
Fasting serum TMAO, choline, betaine and carnitine before (pre) and after (post) both the crossover intervention arms (MED-CONTROL and MED-RED). The notations a and b indicate significant differences identified using linear mixed models when there was a group × time interaction, followed by Tukey’s multiple comparison test. Values are median with their interquartile range. ‘a’: *P* = 0·025, difference between pre- *v*. post-Med200 in TMAO. ‘b’: *P* < 0·001, difference between post-Med200 *v*. Med500 in TMAO. TMAO, trimethylamine N-oxide.

Figure 1 depicts serum fasting TMAO, choline, betaine and carnitine concentrations at pre- (baseline) and post-intervention for both MED-CONTROL and MED-RED intervention periods. Serum TMAO was lower post-intervention following the MED-CONTROL period compared with pre-intervention (see Table 1 for values). Post-intervention serum TMAO concentration was lower in MED-CONTROL than in MED-RED. Serum cystatin-C was not a significant covariate when evaluating the effect of the intervention on serum TMAO concentrations. There was no interaction of sex with treatment in any outcome variable. Sex was a significant factor in betaine, men had higher betaine than women irrespective of time or treatment. The difference between vascular age and biological age of ∼1 year at pre-intervention was increased to ∼3·6 years difference post-intervention, suggesting an improvement in vascular health. Table 2 provides the 95 % CI upper, lower and mean estimates for the outcomes presented in this report.

**Fig. 2.**
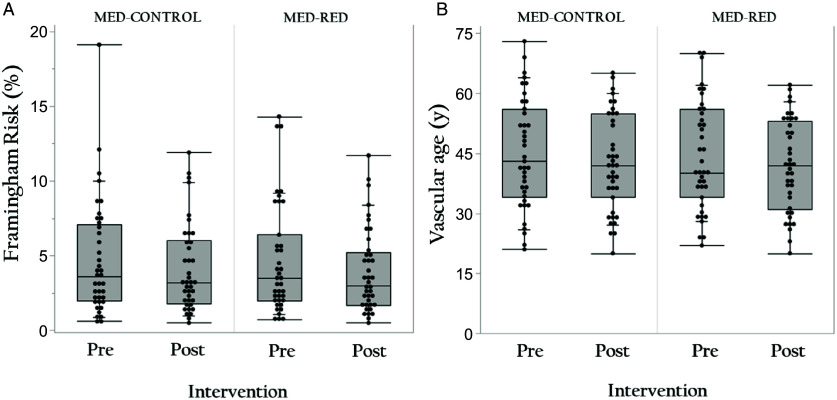
Framingham risk score and vascular age of participants in the study, pre- and post-MED-CONTROL and MED-RED interventions. No significant differences were observed in a linear mixed effect model analysis. There was a 1 % reduction in the Framingham risk % in both the groups between pre- and post-intervention. There was also a 2–3-year reduction in vascular age post-intervention in both treatment arms.

**Table 1.** Circulating trimethylamine N-oxide (TMAO) and its precursor metabolites, along with their linear mixed model *P*-values (Mean values and standard deviations)

		MED-CONTROL				MED-RED				*P* (sex)	*P* (treatment × time interaction)	*P* (sex × treatment interaction)	*P* (pre- *v*. post-MED-CONTROL)	*P* (pre- *v*. post-MED-RED)	*P* (post-MED-CONTROL *v*. post-MED-RED)
		Mean	sd	Mean	sd	Mean	sd	Mean	sd						
		Pre		Post		Pre		Post							
TMAO (µm)	All (*n* 41)	5·2	0·8	3·1	0·2	4·4	0·5	5·0	0·5	0·169	0·013	0·331	0·025	0·537	< 0·001
	Female (*n* 27)	5·6	4·8	3·3	1·1	4·2	1·9	5·0	1·9						
	Male (*n* 12)	3·9	2·0	3·0	1·2	4·7	3·1	4·6	3·2						
Choline (µm)	All (*n* 41)	5·7	0·2	5·8	0·2	6·1	0·3	6·3	0·4	0·930	0·586	0·100	–	–	–
	Female (*n* 27)	5·9	1·4	5·8	1·4	5·9	1·5	6·3	2·2						
	Male (*n* 12)	5·4	1·1	5·7	1·4	6·6	1·5	6·2	1·8						
Betaine (µm)	All (*n* 41)	34·4	2·4	37·6	2·2	33·3	2·3	36·5	2·8	0·016	0·876	0·768	–	–	–
	Female (*n* 27)	32·8	13·4	34·7	11·3	31·5	11·6	33·3	12·6						
	Male (*n* 12)	38·4	12·2	43·4	14·9	38·8	9·7	43·1	14·9						
Carnitine (µm)	All (*n* 41)	42·0	1·8	42·3	1·5	41·3	1·6	44·8	2·0	0·162	0·298	0·278	–	–	–
	Female (*n* 27)	40·5	9·9	40·5	7·8	39·1	6·2	41·9	7·8						
	Male (*n* 12)	45·4	8·6	46·4	9·1	47·0	8·7	49·8	12·6						

“–” Not done because interaction term was not significant.

**Table 2.** 95 % confidence interval mean estimate, lower and upper interval limits of primary parameters

Intervention	Parameter	Pre-intervention			Post-intervention		
		Lower CI	Estimate	Upper CI	Lower CI	Estimate	Upper CI
MED-CONTROL	Serum betaine (µm)	29·5	34·4	39·3	33·0	37·6	42·1
	Serum carnitine (µm)	38·3	42·0	45·6	39·3	42·3	45·3
	Serum choline (µm)	5·23	5·72	6·20	5·27	5·76	6·24
	Serum TMAO (µm)	3·62	5·19	6·77	2·74	3·14	3·53
	FRS (%)	3·48	4·72	5·96	3·12	4·07	5·03
	Vascular age (years)	40·5	44·9	49·4	38·8	42·7	46·6
	Vascular age (years) – biological age (years)	–3·34	–1·00	1·34	–5·34	–3·21	–1·07
MED-RED	Serum betaine (µm)	28·6	33·3	38·0	30·7	36·5	42·2
	Serum carnitine (µm)	38·0	41·3	44·5	40·7	44·8	49·0
	Serum choline (µm)	5·50	6·13	6·75	5·51	6·35	7·19
	Serum TMAO (µm)	3·44	4·39	5·33	4·06	5·02	5·98
	FRS (%)	3·44	4·65	5·87	3·01	3·93	4·86
	Vascular age (years)	40·1	44·5	48·9	38·2	42·0	45·7
	Vascular age (years) – biological age (years)	–3·96	–1·44	1·07	–6·14	–3·95	–1·76

FRS, Framingham risk score; MED-RED, Mediterranean diet with 500 g red meat/week; MED-CONTROL, Mediterranean diet with 200 g red meat/week; TMAO, trimethylamine N-oxide.

### Association between trimethylamine N-oxide and clinical parameters

To identify associations between the effect of the intervention on TMAO and related metabolites with clinical outcomes, we conducted exploratory analyses. TMAO was not significantly associated with plasma TAG, total cholesterol, HDL-cholesterol or LDL-cholesterol (online Supplementary Table S1). Outcomes from the mixed model ANCOVA to identify covariates of HOMA-IR, HOMA-B, FRS and Framingham risk vascular age are summarised in Table 3. TMAO was a positive covariate when evaluating HOMA-IR, while choline was an inverse covariate. Carnitine was a positive covariate in predicting FRS scores. Betaine, carnitine and choline were also positive covariates predicting vascular age. TMAO, choline, carnitine or betaine were not significant covariates of HOMA-B. It is important to note here that the outcome variables in these models were not significantly different by treatment or time, nor was there an interaction between the two. Hence, these covariates are associated with the outcome variables, irrespective of the amount of red meat consumed.

**Table 3.** Mixed model ANCOVA outcomes for serum trimethylamine N-oxide (TMAO) and related metabolites. (Mean values and 95 % confidence intervals)

Outcome variable	Covariate	Estimate	95 % CI		*P*
			Lower	Upper	
FR score	TMAO	0·037	–0·009, 0·084		0·110
	Choline	0·028	–0·021, 0·077		0·254
	Carnitine	0·082 *	–0·023, 0·041		0·024 *
	Betaine	0·064	–0·014, 0·141		0·107
FR vascular age	TMAO	0·051	–0·024, 0·128		0·185
	Choline	0·068*	–0·001, 0·137		0·051 *
	Carnitine	0·141*	0·045, 0·237		0·004 *
	Betaine	0·125*	0·025, 0·224		0·014 *
HOMA-B	TMAO	0·069	–0·114, 0·252		0·451
	Choline	–0·073	–0·283, 0·137		0·492
	Carnitine	0·084	–0·084, 0·252		0·325
	Betaine	–0·066	–0·255, 0·124		0·494
HOMA-IR	TMAO	0·100*	0·007, 0·194		0·036 *
	Choline	–0·111*	–0·208, –0·012		0·027 *
	Carnitine	0·011	–0·128, 0·150		0·877
	Betaine	–0·142 *	–0·287, 0·002		0·053

FR, Framingham risk; TMAO, trimethylamine N-oxide; HOMA-B, homoeostatic model assessment of pancreatic beta cell function; HOMA-IR, homoeostatic model assessment of insulin resistance.

* indicates significant covariates.

## Discussion

This research identified that TMAO was lower in the MED-CONTROL treatment post-intervention compared with MED-RED. Thus, a lower intake of lean red meat in the context of a MED-EP led to a reduced concentration of circulating TMAO, the primary hypothesis motivating this study. Exploratory analysis identified relationships between HOMA-IR and TMAO using mixed model ANCOVA, irrespective of group. The primary study identified a 1 % risk reduction in the 10-year lipid-based FRS score following a relatively short 5-week intervention, irrespective of the amount of red meat^(13)^. Mixed model ANCOVA approaches identified a positive association between TMAO and the insulin resistance surrogate HOMA-IR, and an inverse association between betaine and HOMA-IR. There was a 2–3-year reduction in the vascular age in both periods, which translated to a larger difference (–3·6 years) between vascular age and biological age. Vascular age, while often used as an educational tool by clinicians to inform patients about their cardiovascular health status, also sometimes identifies risks that are not captured by the FRS^(22)^.

TMAO is implicated in CVD risk by several epidemiological studies in diverse populations^(23–25)^. In a large parallel design RCT testing the effect of a no-meat diet to a white or red meat diet, with over 110 participants, TMAO concentrations reported ranged from < 0·1 µm to > 50 µm, suggesting high inter-individual variability. The mean TMAO concentration was ∼3–4 µmol in subjects consuming the white or no meat intervention but was 3-fold elevated, ∼10 µm, in subjects consuming a red meat diet^(12)^. There was no detectable difference between the white meat and non-meat interventions. In comparison to patients with the highest risk of CVD, TMAO concentrations in the present study were moderately low (4·4 µm average)^(26,27)^. Thus, the TMAO concentrations in the present study are similar to values previously reported in studies utilising healthy subjects where median values < 4·0 µm^(12,28)^. Unlike the present study and those of Wang *et al*.^(12)^ that focused on red meat, diet interventions using eggs have failed to demonstrate that increased dietary choline will raise TMAO in healthy participants. Lemos *et al*. reported a fasting plasma TMAO value of 3 µm post-intervention^(29)^. Similarly, Zhu *et al*. who reported TMAO concentrations of 2·3 µm in postmenopausal women, and in both studies, TMAO concentrations were unaffected by egg supplementation^(30)^. An important consideration is the clinical significance of the reduction in TMAO. Circulating concentrations of TMAO have been reported to be > 7·9 µm in individuals with the highest risk of CVD, in patients with coronary artery disease^(27)^. Translating the reduction in TMAO observed in this study and the former study by Wang *et al*.^(12)^ is difficult as diet interventions focus on generally healthy individuals and are relatively short as compared with the development of CVD.

Primary prevention of CVD often involves dietary modifications to reduce risk factors such as plasma LDL or blood pressure. In the case of TMAO, several foods are rich sources of its precursors including fish^(31)^, eggs^(29)^, dairy product^(32)^ and red meat^(28)^. Red meat and eggs have been implicated in increased CVD risk by increasing plasma TMAO concentrations^(12)^, while vegan and vegetarian diets have been associated with low TMAO concentrations and lower CVD risk^(33,34)^. To our knowledge, the present study is the first to assess the effect of a 5-week MED-EP intervention with varying amounts of red meat in adults on downstream effects in TMAO and related metabolites. Several studies have replaced red meat with chicken (or compared vegans to omnivores) to demonstrate that TMAO can be lowered using dietary modifications that eliminate red meat intake^(12,33,34)^. Our results indicate that reducing instead of eliminating red meat consumption can also lower TMAO concentrations. Whether this TMAO-lowering effect is specific to the combination of a MED-EP along with lowered red meat remains to be tested.

The biological pathway by which dietary components affect TMAO concentration is complex and is influenced by genetic and microbial factors that impact the conversion of dietary precursors to TMAO^(35,36)^. A MED-EP, as mentioned earlier, has been shown to reduce circulating TMAO^(37)^. In the present study, TMAO concentration was reduced following the MED-CONTROL intervention. This effect was not observed in the MED-RED intervention. This suggests that the lower red meat intake of 200 g/week along with the Mediterranean diet is capable of reducing this indicator of CVD risk, while the 500 g/week does not have the same effect. However, none of the TMAO metabolic precursors was changed by the MED-EP diets. This suggests that other aspects of TMAO metabolism we did not measure, such as microbiota composition^(38)^, *FMO3* genotype^(39)^ or other novel dietary factors such as trimethyllysine^(40)^ or 3, 3-dimethyl-1-butanol (DMB)^(41)^, could also influence TMAO concentrations. Alternatively, this lends support to the notion that our understanding of TMAO, its metabolism and its downstream health effects are incomplete.

It is important to note that the primary study suggests the MED-RED treatment has cardio-protective effects mainly through reducing plasma total and LDL-cholesterol concentrations^(13)^. Contrastingly, the present study suggests that the MED-CONTROL, but not MED-RED treatment, reduced TMAO. CVD risk is polygenic^(42)^ and involves several mechanisms resulting in diseases such as dyslipidemia and endothelial dysfunction. These data further strengthen the notion that dietary effects on cardiovascular risk are complicated and not uniform.

Using exploratory models, this study also identified a positive association between TMAO and HOMA-IR, a surrogate for insulin resistance. HOMA-IR is commonly used in both clinical and epidemiological settings^(17)^. Interestingly, irrespective of group, our mixed model ANCOVA identified TMAO to co-vary with HOMA-IR. Although one should interpret these exploratory analyses with caution, they are congruent with several published reports. Epidemiological studies have linked high circulating TMAO concentrations with a higher risk for T2D and the metabolic syndrome^(43–45)^. The ORIGINS (Oral Infections, Glucose Intolerance and Insulin Resistance) study with 300 men and women without diabetes identified a positive association between TMAO and prevalence of prediabetes, but not insulin resistance^(46)^. A similar association between circulating TMAO and T2D risk was also observed in the PREDIMED study^(47)^. While no clear mechanism has been identified, a recent Mendelian randomisation analysis suggests that elevated circulating TMAO is a consequence of T2D and not causal^(48)^. What is not clear from genetic approaches such as Mendelian randomisation is the role of specific dietary components and eating patterns to modulate the relationship between TMAO and HOMA-IR. Given epidemiological and clinical evidence, albeit associative and not causal, the relationship between TMAO and HOMA-IR needs further study.

### Strengths and limitations

Since it is a secondary analysis, this study was not powered to detect differences in these outcomes. However, this controlled feeding trial did perturb TMAO concentrations differently during the two periods of the intervention. While it is true that the primary study was not powered to detect differences in intervention-induced responses, the current results provide potentially important data to estimate sample size for future *a priori*, hypothesis-driven studies. The exploratory analyses were designed not necessarily to confirm observations made from the models but rather to propose that these associations warrant further attention and inquiry.

### Conclusions

We report here that a MED-EP with 200 g/week red meat was able to reduce serum TMAO, as hypothesised, compared with a MED-EP with 500 g/week red meat. Further, TMAO and HOMA-IR displayed positive covariance, suggesting clinically relevant metabolic risk implications of TMAO. Future longer-term interventions are needed to investigate whether these differences are sustainable or increase over time.

## Supplementary material

For supplementary material accompanying this paper visit https://doi.org/10.1017/S0007114521004694. click here to view supplementary material
