# Supplementary material for: Adopting a Mediterranean-style eating pattern with low, but not moderate, unprocessed, lean red meat intake reduces fasting serum trimethylamine N-oxide (TMAO) in adults who are overweight or obese
Source: Br J Nutr. 2021 Nov 26;128(9):1738–46. doi: 10.1017/S0007114521004694 (PMC9133270; doi:10.1017/S0007114521004694)
Supplement: Supplementary file 1 [file S0007114521004694sup001.docx]

**Supplemental Table 1**: Mixed model analysis of covariance outcomes for serum TMAO and related metabolites. Bolded text indicates significant covariates.

| **Outcome variable** | **Covariate** | **Estimate** | **95% CI** | | **p value** |
| --- | --- | --- | --- | --- | --- |
|  |  |  | **Lower** | **Upper** |  |

| **Total Cholesterol** | TMAO | 0.062 | -0.031 | 0.156 | 0.190 |
| --- | --- | --- | --- | --- | --- |
|  | Choline | 0.080 | -0.021 | 0.180 | 0.119 |
|  | Carnitine | 0.078 | -0.060 | 0.214 | 0.268 |
|  | Betaine | -0.028 | -0.171 | 0.115 | 0.701 |
|  |  |  |  |  |  |
| **LDL-Cholesterol** | *TMAO* | *0.096* | *-0.006* | *0.199* | *0.067* |
|  | Choline | 0.070 | -0.038 | 0.179 | 0.201 |
|  | **Carnitine** | **0.166** | **0.023** | **0.309** | **0.023** |
|  | Betaine | 0.039 | -0.110 | 0.189 | 0.603 |
|  |  |  |  |  |  |
| **HDL-cholesterol** | TMAO | 0.037 | -0.034 | 0.106 | 0.301 |
|  | **Choline** | **0.076** | **0.006** | **0.145** | **0.033** |
|  | Carnitine | -0.010 | -0.104 | 0.083 | 0.821 |
|  | Betaine | 0.017 | -0.087 | 0.122 | 0.745 |
|  |  |  |  |  |  |
| **Triglycerides** | TMAO | 0.037 | -0.043 | 0.115 | 0.360 |
|  | Choline | 0.036 | -0.048 | 0.119 | 0.398 |
|  | Carnitine | -0.046 | -0.156 | 0.064 | 0.410 |
|  | Betaine | -0.066 | -0.179 | 0.047 | 0.251 |

TMAO – Trimethylamine oxide. Significant associations in **Bold.**
